# Supplementary material for: Effects of Dispersant on the Petroleum Hydrocarbon Biodegradation and Microbial Communities in Seawater from the Baltic Sea and Norwegian Sea
Source: Microorganisms. 2023 Mar 29;11(4):882. doi: 10.3390/microorganisms11040882 (PMC10142239; doi:10.3390/microorganisms11040882)
Supplement: Supplementary file 1 [file microorganisms-11-00882-s001.zip › microorganisms-2222159-supplementary.pdf]

Supplementary material

Table S1. Petroleum hydrocarbon (C10-C40) concentrations of different WAF (without dispersant) and CE-WAF (with dispersant) dilutions in the control experiment with GoF seawater. Averages are means of 2, in abiotic controls means of 3.

Control experiment treatments	Petroleum hydrocarbons C10-C40 (µg/L)	
	Average	Stdev
Undiluted WAF 0h	1020	180
Undiluted CEWAF 0h	555000	45000
WAF 1:1 0h	505	45
WAF 1:1 12d	305	5
WAF 1:1 abiotic control 12d	363	91
CEWAF 1:50 0h	10000	0
CEWAF 1:50 12d	11500	1500
CEWAF 1:50 abiotic control 12d	10700	1203
Only dispersant 1:50 0h	745	25
Only dispersant 1:50 12d	565	115

Table S2. PAH concentrations of different WAF (without dispersant) and CE-WAF (with dispersant) dilutions in the control experiment. Averages are means of 2. For calculation of PAH sum and averages values below detection limit have been set to half of the detection limit.

PAH compounds	PAH concentrations (µg/L)					
	Undiluted WAF 0h	Undiluted CEWAF 0h	WAF 0h (1:1 dilution)	WAF 12d (1:1 dilution)	CEWAF 0h (1:50 dilution)	CEWAF 12d (1:50 dilution)
PAH Sum	388.76±50.41	4819.3±566.69	173.92±22.31	6.82±0.62	158.71±17.5	59.57±6.73
1-Methylnaphthalene	51.5±1.5	940±20	24±2	1.725±1.375	30±3	18.5±2.5
2-Methylnaphthalene	125±5	2400±100	54±3	0.905±0.795	73±8	21.5±0
Anthracene	0.0945±0.0025	1.5±0	0.0515±0.0015	0.054±0.003	0.155±0.015	0.16±0
Acenaphthene	1.2±0	78±52	0.61±0.01	0.855±0.445	3.75±1.05	3.05±0.35
Acenaphthylene	0.525±0.095	104.5±5.5	0.315±0.005	0.655±0.275	2.6±1.2	1.7±0.1
Benzo(a)anthracene	0.0945±0.0025	0.005±0*	0.0515±0.0015	0.054±0.003	0.155±0.015	0.160
Benzo[a]pyrene	0.005±0*	1.85±0.65	0.005±0*	0.005±0*	0.049±0.023	0.053±0.001
Benzo[b]fluoranthene	0.005±0*	2.95±1.45	0.005±0*	0.005±0*	0.116±0.054	0.12±0
Benzo[e]pyrene	0.005±0*	3.75±2.15	0.005±0*	0.005±0*	0.166±0.074	0.175±0.005
Benzo[ghi]perylene	0.005±0*	1.75±0.75	0.005±0*	0.005±0*	0.057±0.026	0.063±0.002
Benzo[k]fluoranthene	0.005±0*	3.2±1.7	0.005±0*	0.005±0*	0.116±0.054	0.12±0
Dibenz[a.h]anthracene	0.005±0*	0.945±0.055	0.005±0*	0.005±0*	0.0085±0.0035	0.005±0*
Phenanthrene	2.3±0	77.5±42.5	1.2±0	1.05±0.05	6.1±1.2	1.6±0.1
Fluoranthene	0.034±0	0.00415±0.00165	0.0205±0.0005	0.021±0.001	0.355±0.125	0.32±0.01
Fluorene	3±0.1	185±5	2.1±0	1.15±0.05	5.8±1.2	4.7±0
Indeno[1.2.3-cd]pyrene	0.005±0*	1.19±0.31	0.005±0*	0.005±0*	0.0225±0.0105	0.014±0.009
Chrysene	0.012±0	5.7±3.5	0.0063±0	0.005±0*	0.245±0.105	0.25±0
Naphthalene	205±15	1000±0	91.5±3.5	0.308±0.242	35±2	6.1±4.9
Perylene	0.005±0*	3±1.5	0.005±0*	0.005±0*	0.123±0.057	0.125±0.005
Pyrene	0.0025±0*	0.0025±0*	0.0025±0*	0.0025±0*	0.0025±0*	0.0025±0*
Triphenylene	0.0165±0.0015	8.45±5.55	0.05±0*	0.0305±0.0195	0.375±0.155	0.355±0.005

*Measurements below LOD (value divided by 2).

Table S3. Biomarker ratios (Pristane/Phytane, C17/C18, C17/Pristane, C18/Phytane, Norpristane/Pristane) for 12d CE-WAF, 12d sterile control CE-WAF and North Sea Crude oil. Samples were obtained from the control experiment.

Sample/Treatment*	Pr/Pf	C17/C18	C17/Pr	C18/Pf	NPr/Pr
North Sea Crude oil	1.14	0.54	1.88	3.98	1.04
12d sterile control CE-WAF	1.19	0.53	1.78	3.98	1.00
12d CE-WAF	0.98	0.50	2.25	4.38	1.19

*12d WAF and 12d sterile control WAF were also analysed but did not contain these long-chain biomarkers and ratios could thus not be calculated.

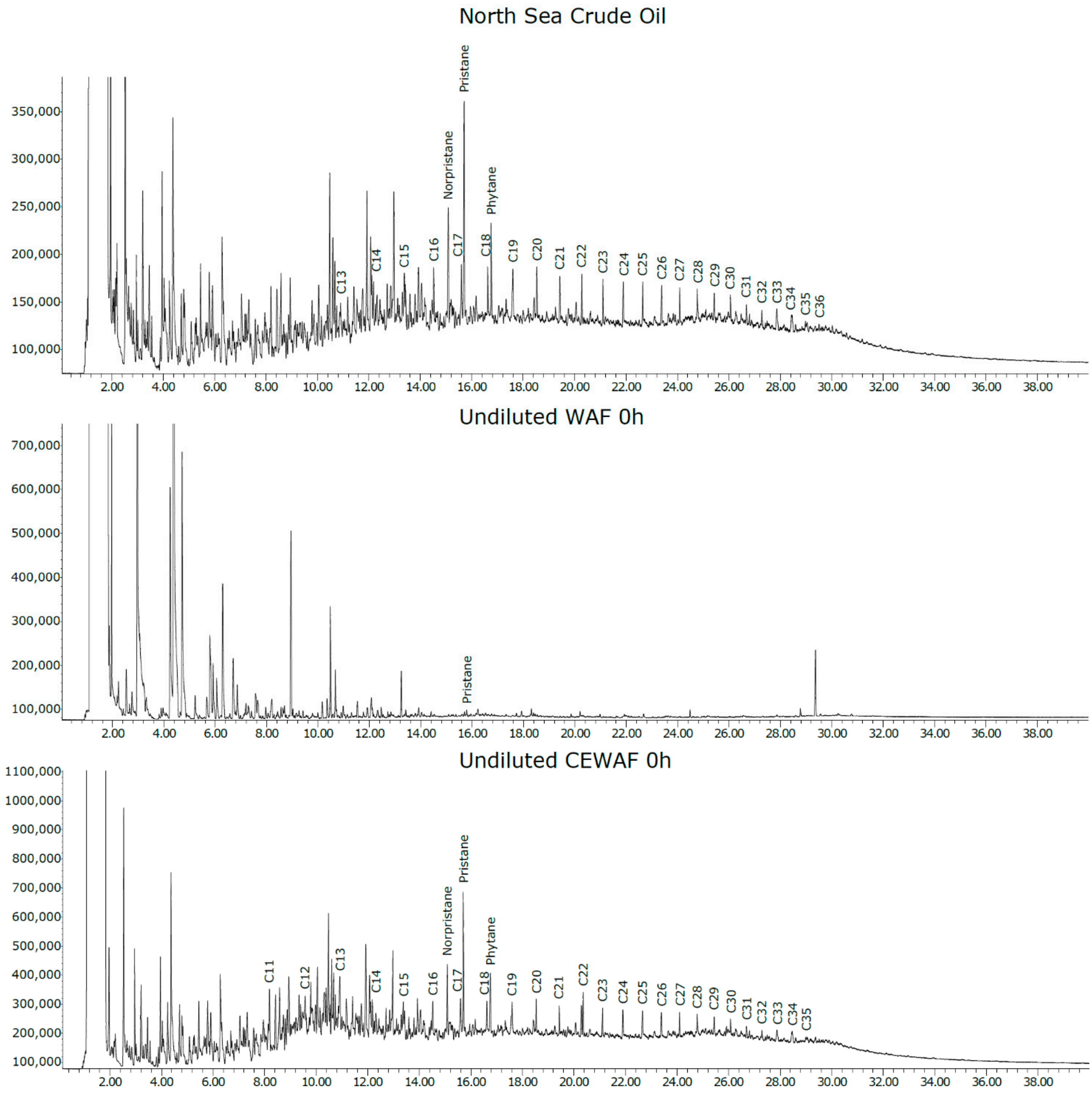

Figure S1. Gas chromatograms for pure North Sea crude oil, undiluted CE-WAF (with dispersant) 0h and undiluted WAF (without dispersant) at the start of the control experiment

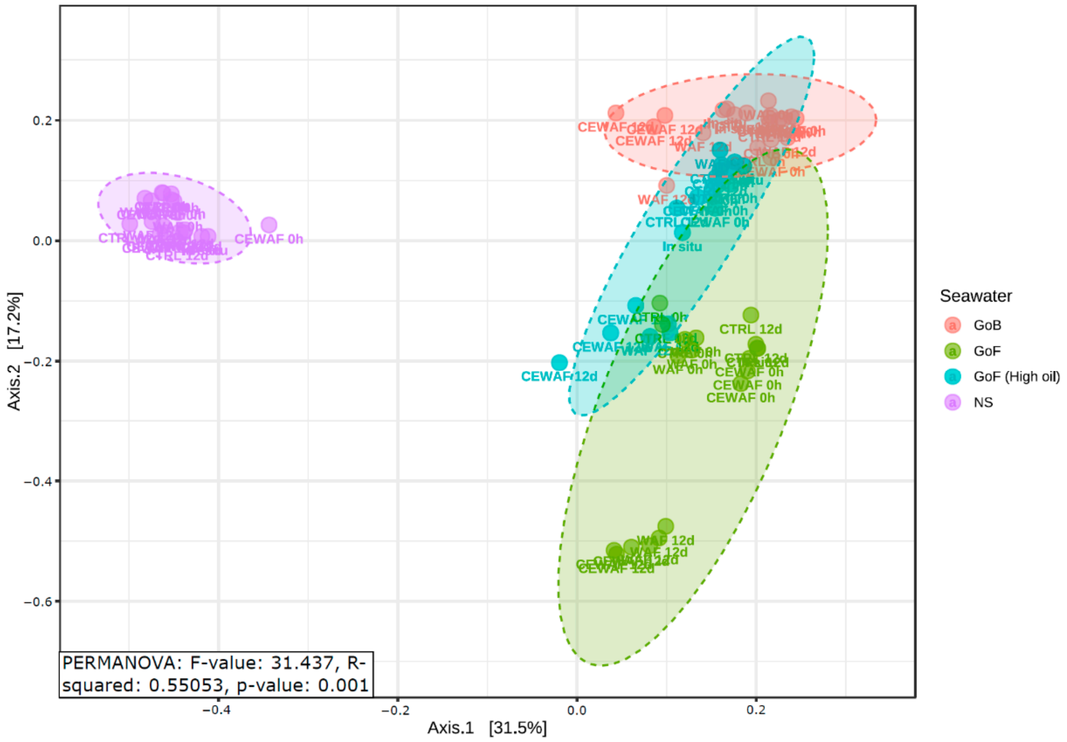

Figure S2. Principal Coordinates Analysis (PCoA) of microbial communities comparing different microcosm experiments. PCoA was based on Bray-Curtis distance matrix using OTU level data.

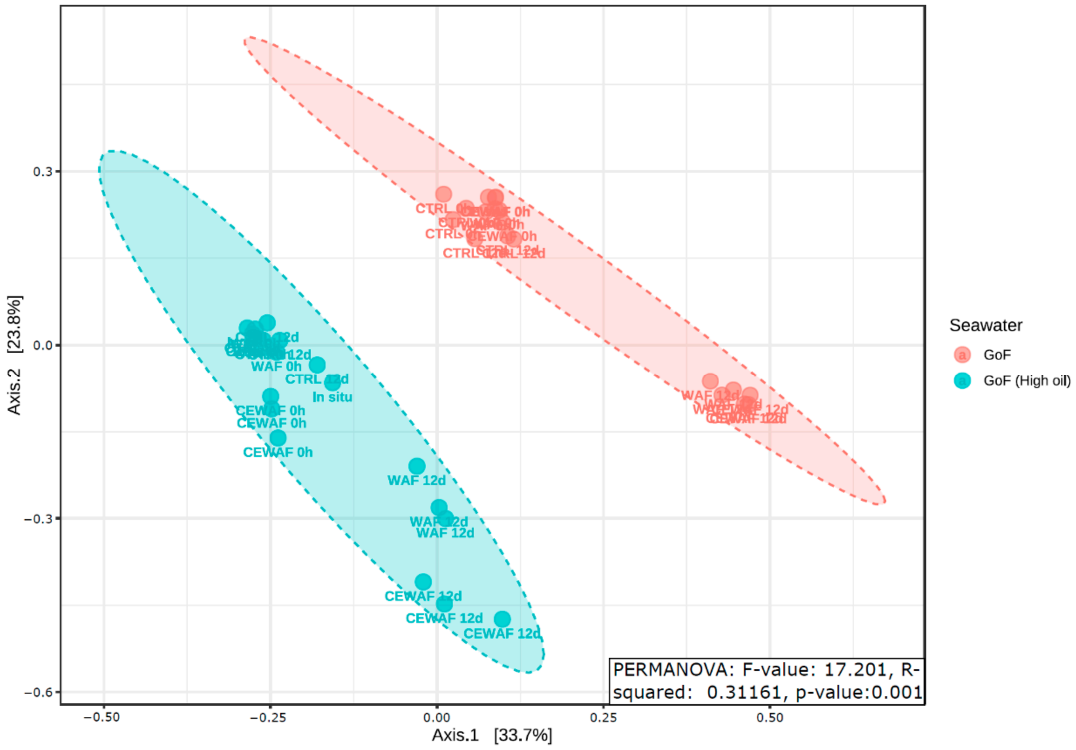

Figure S3. PCoA of microbial communities comparing high and low oil level Gulf of Finland experiments. PCoA was based on Bray-Curtis distance matrix using OTU level data

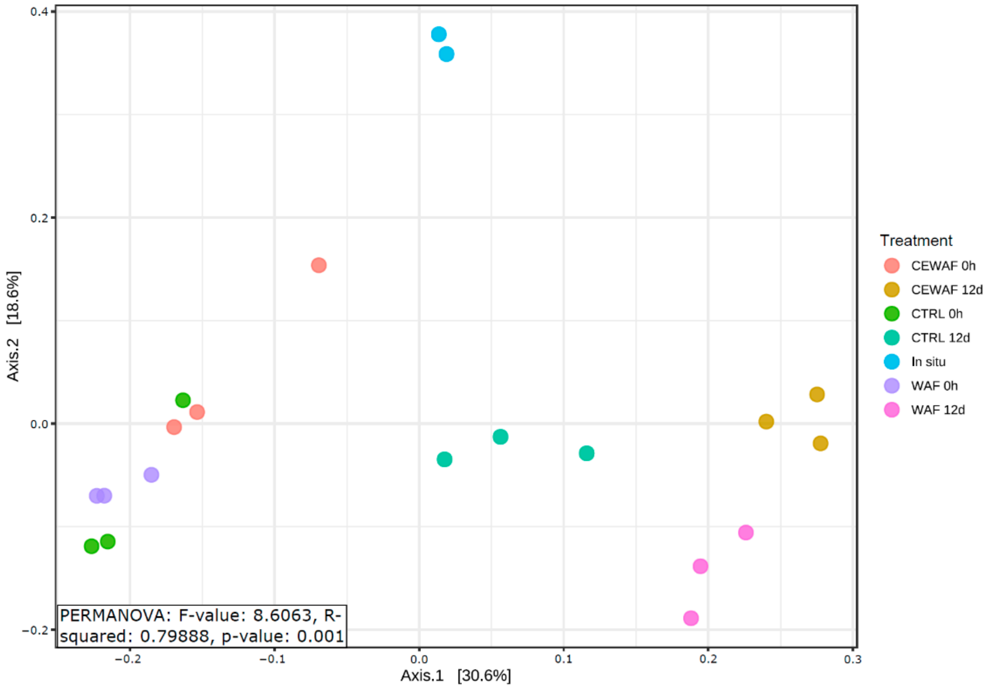

Figure S4. PCoA of microbial communities of Norwegian Sea experiment. PCoA was based on Bray-Curtis distance matrix using OTU level data.

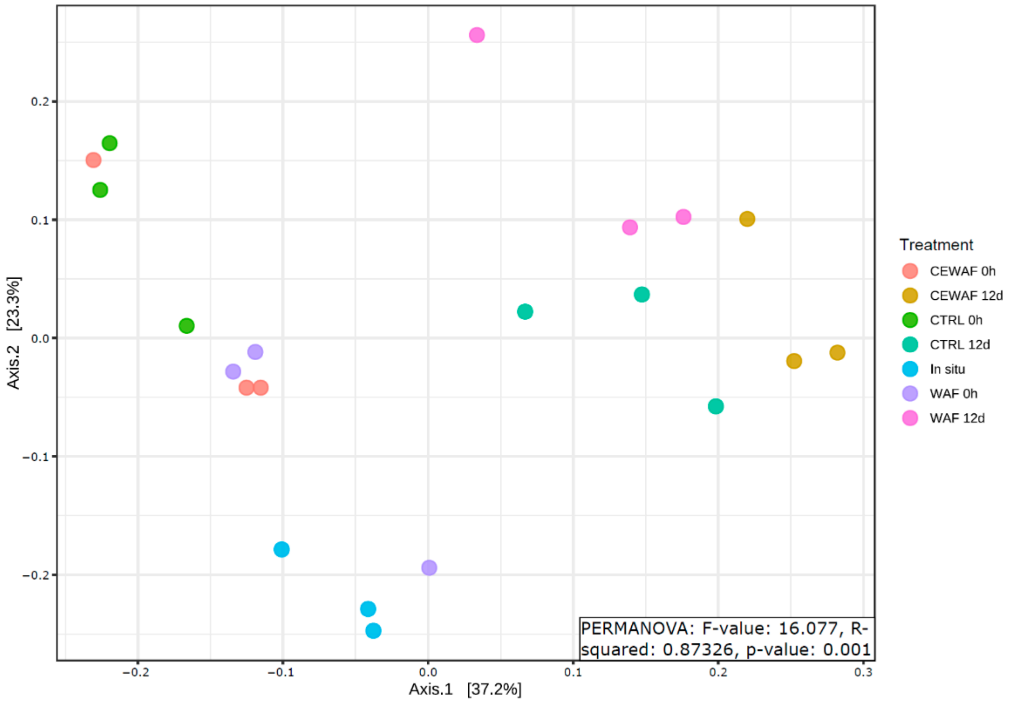

Figure S5. PCoA of microbial communities of Gulf of Bothnia experiment. PCoA was based on Bray-Curtis distance matrix using OTU level data.

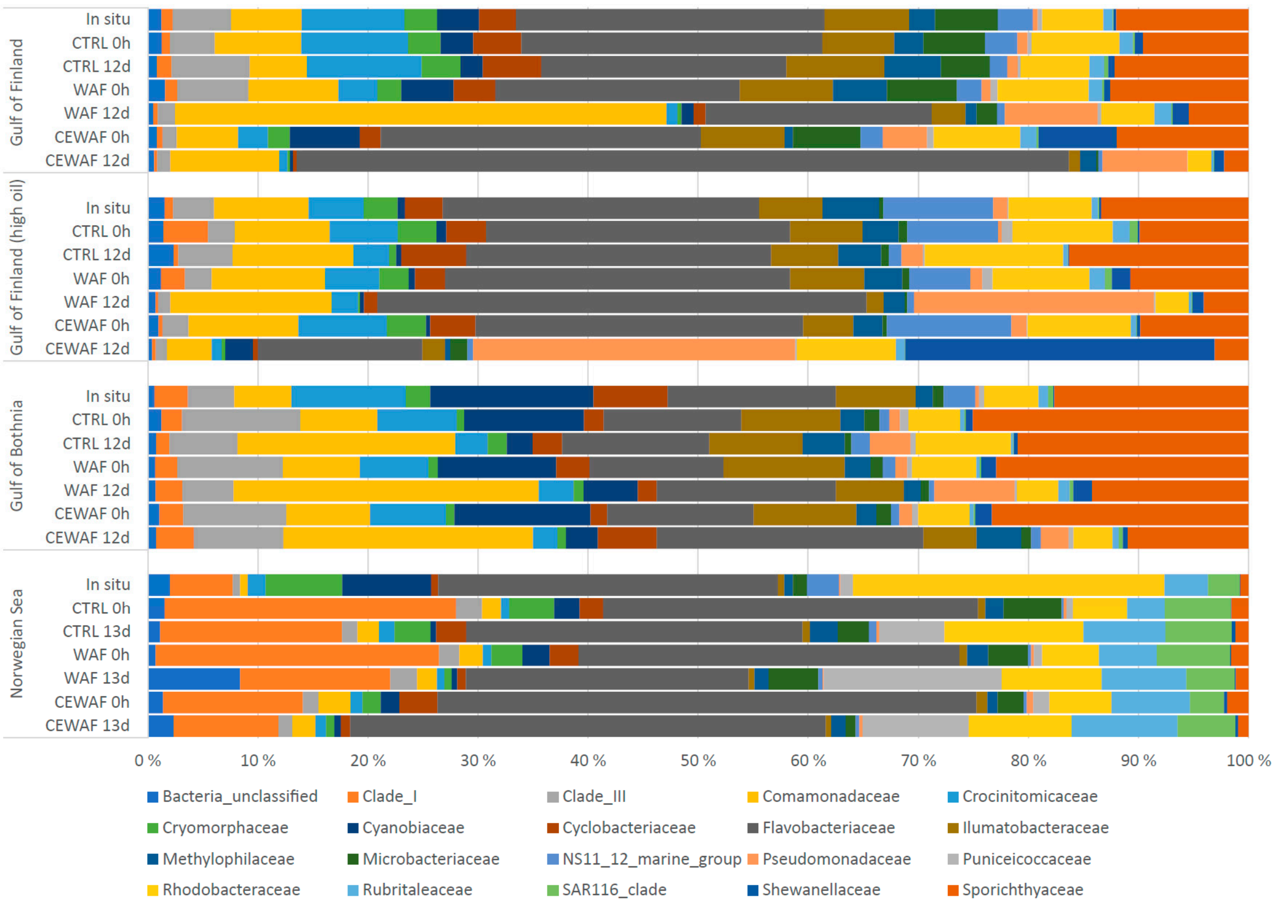

Figure S6. The relative abundance of top 20 most abundant bacterial taxa on family level. Each bar in the figure is the average abundance calculated from three replicates for different experiments (Gulf of Bothnia, Norwegian Sea and Gulf of Finland with high and low oil concentration seawater).
